# Soft Independent Modeling of Class Analogies for the Screening of New Psychoactive Substances through UPLC-HRMS/MS

**DOI:** 10.1021/acs.analchem.5c02450

**Published:** 2025-07-11

**Authors:** Ilenia Bracaglia, Sara Gamberoni, Camilla Montesano, Francesco Bartolini, Sabino Napoletano, Claudio D’Alfonso, Chiara Nieri, Federico Marini, Manuel Sergi

**Affiliations:** † Department of Chemistry, 9311 Sapienza University of Rome, Rome 00185, Italy; ‡ Department of Public Health and Infectious Diseases, Sapienza University of Rome, Rome 00185, Italy; § Department of Public Security, Directorate for the Forensic Science Police and the Cyber Security, Forensic Science Police Service, Rome 00173, Italy

## Abstract

The proliferation of NPS has become a global issue, due to their easy availability and ability to bypass drug screening tests. These substances are particularly concerning because of their unpredictable toxicological effects and the analytical challenge in identifying them. The present study combines advanced analytical strategies based on UPLC-HRMS with multivariate analysis to identify and classify unknown NPS. Tandem mass spectrometry (MS/MS) spectra of 159 analytical standards were acquired, retention times and MS data were preprocessed and organized in separate matrices to obtain a training set (including 75% of the analytes) and a test set (with the remaining 25%). Principal component analysis (PCA) revealed distinct clusters for different NPS classes, such as benzodiazepines, JWH, and PINACA, while others, like cathinones and fentanyl analogues, showed greater dispersion. Subsequently, soft independent modeling of class analogies (SIMCA) classification models were built. The models were validated, achieving optimal values, and correctly classifying analytes included in the test set, especially when considering the data obtained at lower collision energy. External validation was conducted using three real seized drug samples. This confirmed the models’ ability to classify data not included in the training set, as reflected in the positive validation parameters achieved for each model. Although some misclassifications occurred due to the limited availability of standards for certain classes, the SIMCA models proved highly effective in identifying NPS, demonstrating their value as a reliable tool for supporting forensic investigations.

## Graphical abstract

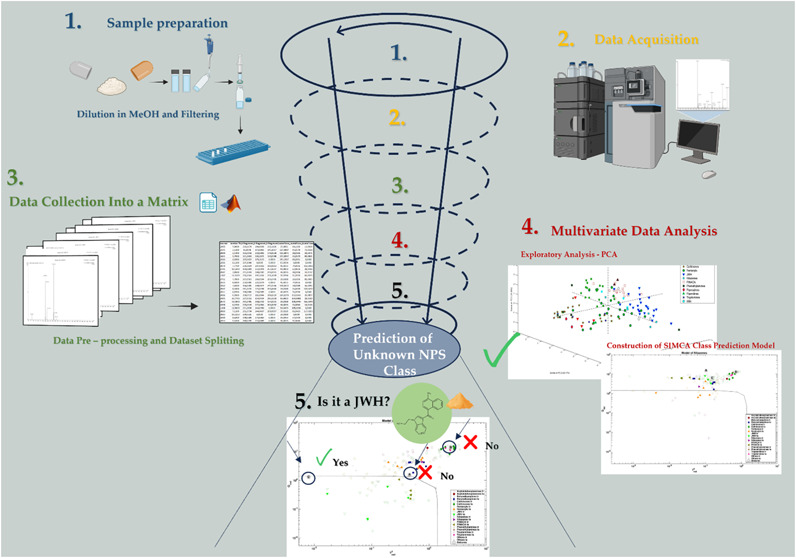

## Introduction

The detection and analytical recognition of New Psychoactive Substances (NPS) represents a current challenge in forensic drug analysis. The spread of these substances has become a global phenomenon, involving more than 800 different compounds, as reported by the United Nations Office on Drugs and Crime (UNODC) through its Early Warning Advisory (EWA).^1^ NPS are defined “substances of abuse, either in a pure form or a preparation, that are not controlled by the 1961 Single Convention on Narcotic Drugs or the 1971 Convention on Psychotropic Substances, but which may pose a public health threat”.^2^ The development and production of NPS began decades ago with the aim of creating pharmacologically active compounds that could replicate the effects of the main categories of internationally controlled psychotropic drugs.^3^ Many of these drugs share common chemical structural features with controlled substances, often differing by only a slight modification of the original molecule.^4^ As a result, the detection and analytical identification of unknown NPS is difficult, since their rapid spread often exceeds existing analytical protocols, and is further complicated by the lack of reference standards.^5^

When dealing with drug seizures, typically an initial qualitative analysis using low cost, portable instruments is carried out; these may identify the characteristic chemical groups of the substances but have low selectivity and specificity.^6^ More sophisticated techniques including nuclear magnetic resonance (NMR), gas chromatography–mass spectrometry (GC-MS), liquid chromatography tandem mass spectrometry (LC-MS/MS)^7,8^ and especially high-resolution mass spectrometry (HRMS) are then crucial for the identification of unexpected drugs.

At present HRMS is the gold standard for NPS detection, given its ability to record accurate mass measurements that provide information about molecular structures;^3^ but the complexity of data obtained limits its routinary application. For this reason, it is necessary to combine top-down and bottom-up approaches^5,9^ to interpret and simplify data with additional support from databases (such as HighResNPS,^10^ SWGDRUG,^11^ mzCloud) and data mining tools for retrospective analysis.^7,8,12,13^ An analytical approach for identifying both known and unpredicted NPS could involve the analysis of diagnostic fragment ions characteristic of each NPS core. This methodology can be particularly useful for preliminary MS screening, especially when comparative confirmation with analytical reference standards is unavailable.^14–16^ Vincenti et al.^17^ employed a molecular networking strategy to group similar compounds or those sharing the same *m*/*z* into clusters based on the similarity of their MS/MS fragments. This approach facilitated the identification of similarities among MS/MS spectra within a data set and enabled the correlation of unknown but related molecules. Ventura et al.^18^ exploited DART-HRMS technology to observe neutral losses typical of the tryptamine class and employed Hierarchical Cluster Analysis (HCA)^19^ for data analysis; the observed clusters were then used to create a supervised classification model (partial least-squares discriminant analysis, or PLS-DA) to support the identification of unknown tryptamine structures. Other classification models have been used to identify the structural characteristics of illicit drugs from MS,^20,21^ chromatographic^22^ or IR data,^23–25^ however, they have never been applied to putatively identify new drugs.

The present study combined analytical and chemometric tools to detect unknown NPS in seizures through UPLC-QTOF-MS analysis. The preliminary step consisted in analyzing 159 NPS standards to obtain chromatographic and MS/MS information. A data matrix, including *m*/*z* of precursor and fragments, retention time, and neutral losses, was then built and was split into training and test sets. Principal component analysis (PCA)^16,26^ was then applied to explore the data, while a class modeling strategy based on Soft Independent Modeling of Class Analogies (SIMCA)^20,27,28^ was employed to create robust models aimed at predicting the class of unknown NPS. The models were validated on the test set and with real seized samples obtained from the Scientific Police Service.

## Experimental Section

### Chemicals and Reagents

The analytical standards of 159 NPS (Table S1) were purchased from commercial suppliers, including Cayman Chemical (Ann Arbor, Michigan) and LGC Standards (Sesto San Giovanni (MI), Italy). Leucine Enkephalin (Leu Enk) was used for lock mass corrections, and the Waters MajorMix was used for mass calibration. Both were purchased from Waters, Milford, MA. Ultrapure water LC-MS grade was obtained by a Milli-Q Plus Millipore (Bedford, MA); ammonium formate was acquired from Waters, Milford, MA; acetonitrile (AcN), methanol (MeOH) and formic acid (HCOOH) were acquired from Biosolve Chimie (France). All analytical standards were stored at −20 °C.

### Working Solutions

A working solution (WS) containing the 159 standards was prepared at 100 ng mL^–1^ in methanol. A solution containing Leu Enk at 400 ng mL^–1^ in AcN:H2O (50:50, v/v), 0.1% HCOOH was also prepared. All WSs were stored at −20 °C.

### UPLC-MS/MS Analysis

Data acquisition was performed using an UPLC-QTOF system consisting of an ACQUITY I-Class UPLC System coupled to a SYNAPT G2-Si HDMS (Waters Corporation, Milford, MA). Instrument control, data acquisition, and initial data processing were performed with MassLynxTM version 4.2. Chromatographic separation was achieved using a C18 column (ACQUITY UPLC HSS C18 Column, 100 Å, 1.8 μm, 2.1 150 mm) maintained at 50 °C with a flow rate of 0.4 mL min^–1^; injection volume was 10 μL. The mobile phases were 5 mM ammonium formate buffer, pH 3.0 (A), and acetonitrile 0.1% HCOOH (B). The chromatographic separation was performed with the following gradient: phase B was held at 13% in the first 0.5 min, then increased linearly to 50% in 10 min. Then, phase B was rapidly changed to 95% in 0.75 min and held for 1.5 min. After 0.25 min, the column was returned to the original ratio within 4 min. The total run time was 16.5 min.

The TOF-MS was operated in positive electrospray ionization mode (Z-SprayTM, Waters) with the following settings: 800 L/h of nitrogen as the nebulization gas at 500 °C, 40 L/h cone gas flow at 120 °C, a capillary voltage of 3 kV and argon as the collision gas. Data were recorded in profile mode using Full Scan and then MS/MS mode. These scans were carried out in Resolution mode (fwhm 20,000) with a mass range from 50 to 700 Da. MS/MS spectra were acquired with CE ramped from 10 to 30 V, with a scan time of 1 s for each time window centered on the retention time of each analyte ± 30 s (see Table S1). Mass calibration was performed daily with the Major Mix.

### Data Matrix Construction and Preprocessing

UPLC-MS/MS data were processed using MATLAB version R2024a (The MathWorks, Natick, MA): PLS Toolbox version 9.3.1 (eigenvector Technologies, Manson, MA) was used for PCA, while SIMCA models were built using in-house written functions (freely available at: https://github.com/RomeChemometrics/Simca). Waters Forensic Toxicology Library and HighResNPS^10^ were used for spectral comparisons.

MS and MS/MS spectral information on 159 NPS belonging to different classes (Figure 1) were compiled into a matrix of eight variables (159 × 8), including the *m*/*z* precursor, retention time, and the three most intense fragments along with their corresponding neutral losses, as reported in Table S1. These fragments were selected using a relative intensity threshold of 10%.

**Figure 1:**
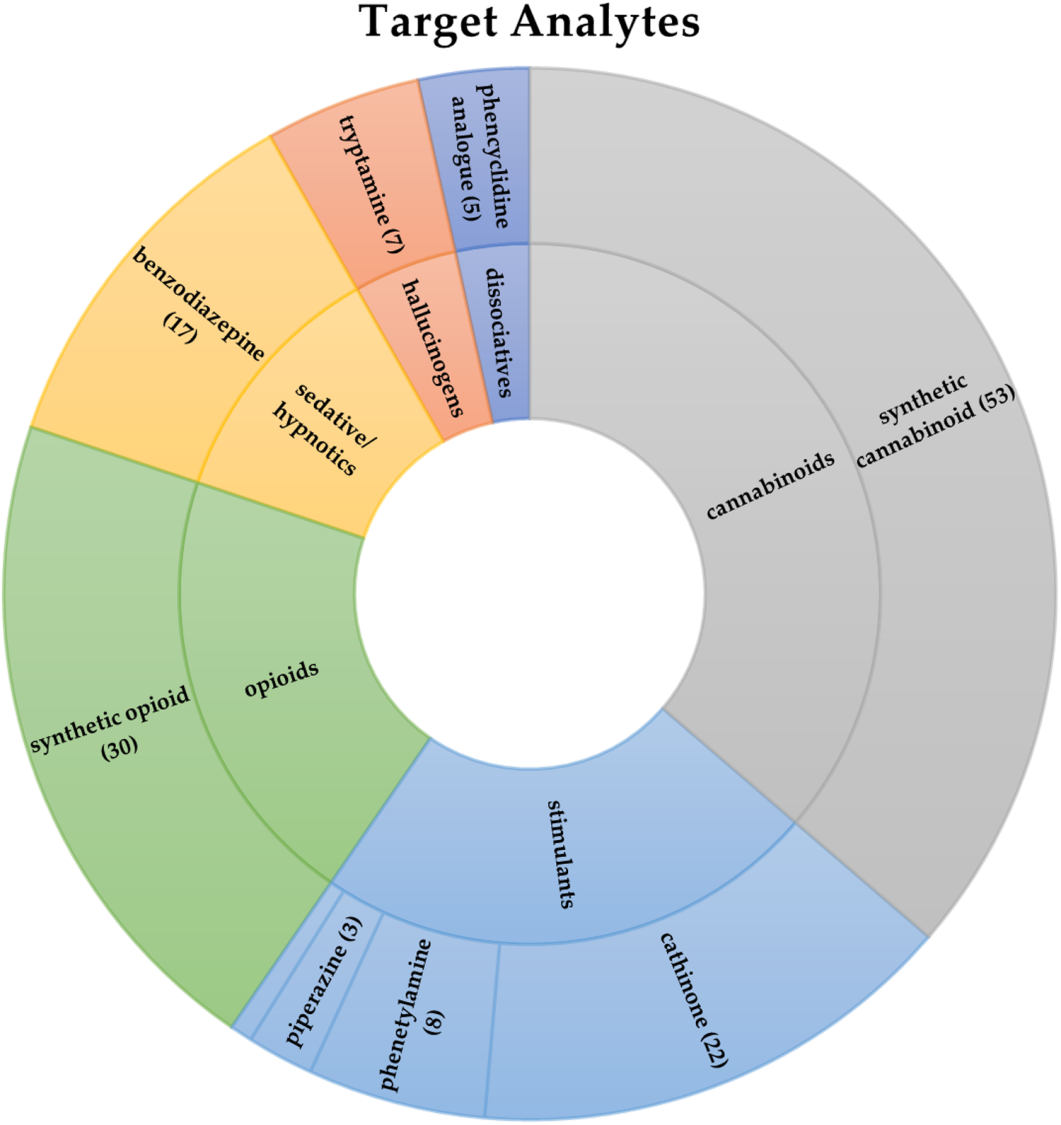
Graphical distribution of analytes included in the target method (159 NPS) according to the classification adopted by EUDA.^30^.

The data set was split into training (75%) and test (25%) sets through a Kennard-Stone algorithm,^29^ in which the training set is used to fit the model, while the test set or validation set is used to check that the trained model works as intended on a set of unknown data. PCA was carried out on the training set data after autoscaling.

### SIMCA Modeling

SIMCA models were built for the most represented groups (benzodiazepines, cathinones, fentanyls, PINACA, and JWH) and four smaller groups (nitazenes, tryptamines, arylcyclohexylamines and phenethylamines) (Table 1).

**Table 1:** Number of Analytes in Each Class for the Construction of the SIMCA Models

drug class	number of analytes	number of principal components
benzodiazepines	17	2
cathinones	22	4
fentanyls	18	4
JWH	17	3
nitazenes	4	2
PINACA	17	1
tryptamines	5	1
arylcyclohexylamines	4	1
phenethylamines	6	2

The number of Principal Components (PCs) was selected, leading to the maximum efficiency (defined as the geometric mean between sensitivity and specificity) in cross-validation. For the models of the most represented groups, the class training data were split into five cancellation groups, while for the four smaller groups, leave-one-out cross-validation was adopted.^31^

The models were first validated on the test set samples.

### External Validation on Real Samples

Three seized drug samples previously identified as containing Androst-3,5-diene-7,17-dione (a steroid, no NPS), 6-APB (phenethylamine), and MDMB-BUTINACA (synthetic cannabinoid) were used for external validation of the SIMCA models. The three samples were labeled as A, B, and C. A and B were in powder form, while C was a plant matrix. The powders were dissolved in methanol at 2 mg/mL, while 5 mg of B was extracted in 1 mL of methanol. All solutions obtained were filtered using Acrodisc Syringe Filter—13 mm, 0.22 μm PTFE filters, then diluted to 0.2 μg/mL and subsequently analyzed by UPLC-HRMS as previously described.

## Result and Discussion

### Data Matrix Building

The analytical standards were selected based on the substances most frequently reported by the UNODC Early Warning Advisory (EWA), the first global monitoring system for NPS.^32^ In this context, the classes with the largest number of substances chosen are synthetic cathinones, synthetic cannabinoids, and synthetic opioids, which mainly include fentanyl analogues.^33,34^

To achieve optimal chromatographic separation and maximize MS spectral response, the chromatographic column, mobile phases, and gradient were carefully selected and tailored, considering the nature of the analytes under examination. The choice of the column and mobile phases was supported by extensive literature studies^35–37^ and experimental testing.

Full Scan and MS/MS acquisition modes were chosen to obtain fragmentation spectra for the analytes under study. The Full Scan analysis of the 159 NPS enabled the determination of characteristic retention times for these substances, allowing for simultaneous gradient adjustments to separate isomeric compounds, such as Ethcathinone, Dimethylcathinone, and 3-Methylmethcathinone, eluting at 1.86, 1.93, and 3.05 min, respectively, as well as α-methyl fentanyl and *cis*-3-methyl fentanyl eluting at 5.41 and 6.64 min, respectively.

Subsequently, MS/MS analysis was performed to obtain specific fragmentation patterns for each analyte, testing two collision energy ramps: 10–30 and 30–50 V. To this aim, the chromatographic run was divided into distinct acquisition time windows of approximately ± 0.7 min. This approach allowed efficient and targeted fragmentation data acquisition across all analytes within the optimized timeframes.

By analyzing the obtained spectra (some examples are shown in Figure 2), it was possible to identify and consider the most intense fragments characteristic of each analyte and compare them with the Waters Forensic Toxicology Library and HighResNPS.^11^ Moreover, the neutral losses were calculated by examining the differences between the signal corresponding to the fragments, ranked in descending order of intensity and above a relative intensity threshold of 10%, and the base peak. This approach provided valuable information about the structure of the compounds, facilitating a deeper understanding of their chemical properties.

**Figure 2:**
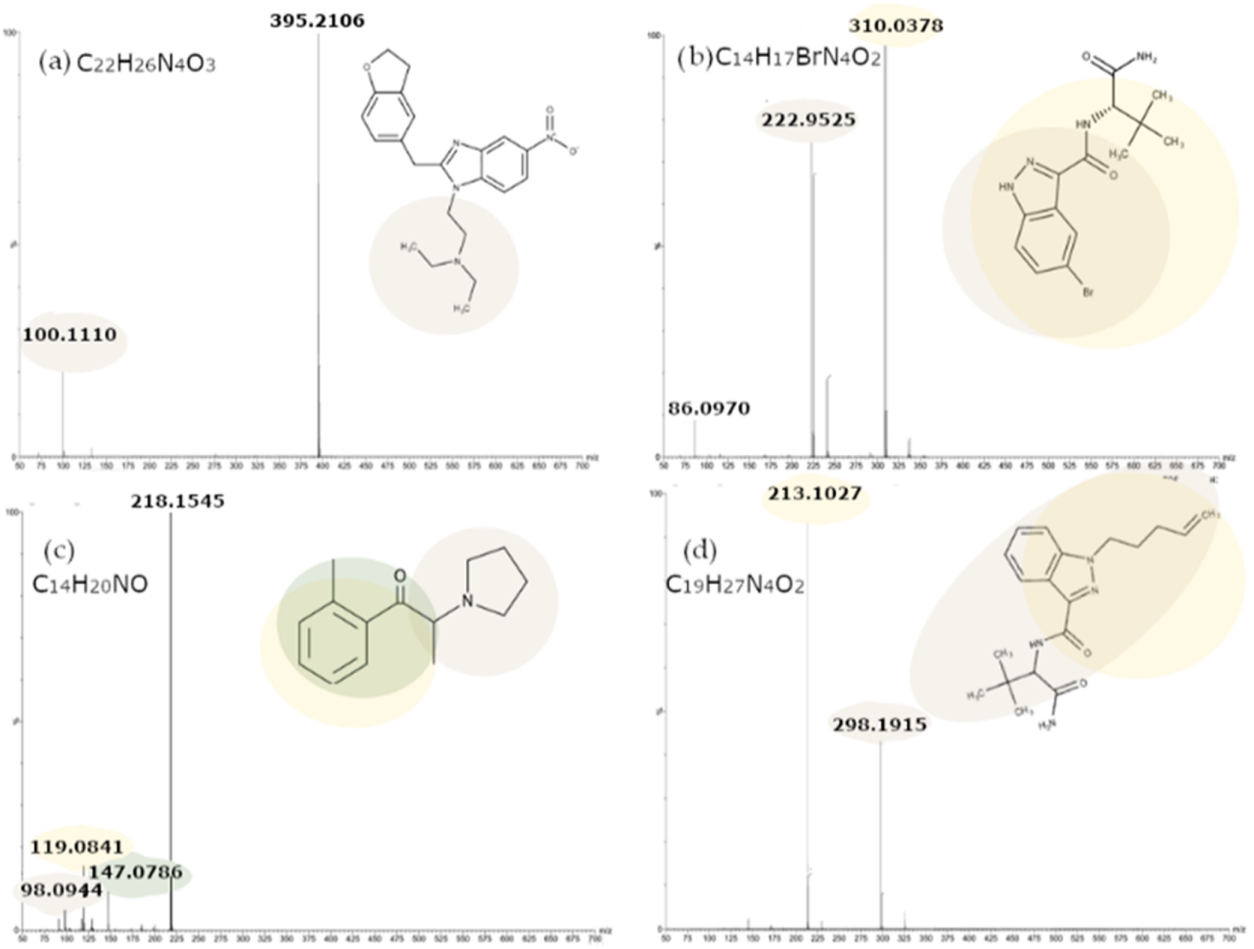
MS/MS spectra (CE ramp of 10–30 V) for: (a) ethylenoxynitazene; (b) ADB-5Br-INACA; (c) 2-Methyl-α-PHP; (d) ADB-4en-PINACA­(+H^+^).

### Data Treatment and Multivariate Data Analysis Strategies

All MS/MS spectral data acquired from the 159 NPS standards, including precursor *m*/*z*, the *m*/*z* values of the three most intense fragments, and the three corresponding neutral losses, were organized within two matrices, the first containing MS/MS spectra acquired in the CE range of 10–30 V (159 × 8), and the second containing MS/MS spectra acquired in the CE range of 30–50 V (142 × 8). These matrices also include chromatographic information such as retention time, which is a characteristic parameter influenced by the physicochemical properties, although it also depends on the chromatographic conditions used.

The preliminary PCA conducted on the training set (122 substances) of the data acquired in the CE range of 10–30 V (Figure 3a,b) already highlighted some well-defined clusters corresponding to specific classes of substances. Only the results of PCA on this matrix are presented, as the exploratory analysis performed on the matrix derived from data acquired in the CE range of 30–50 V did not show any clear groupings.

**Figure 3:**
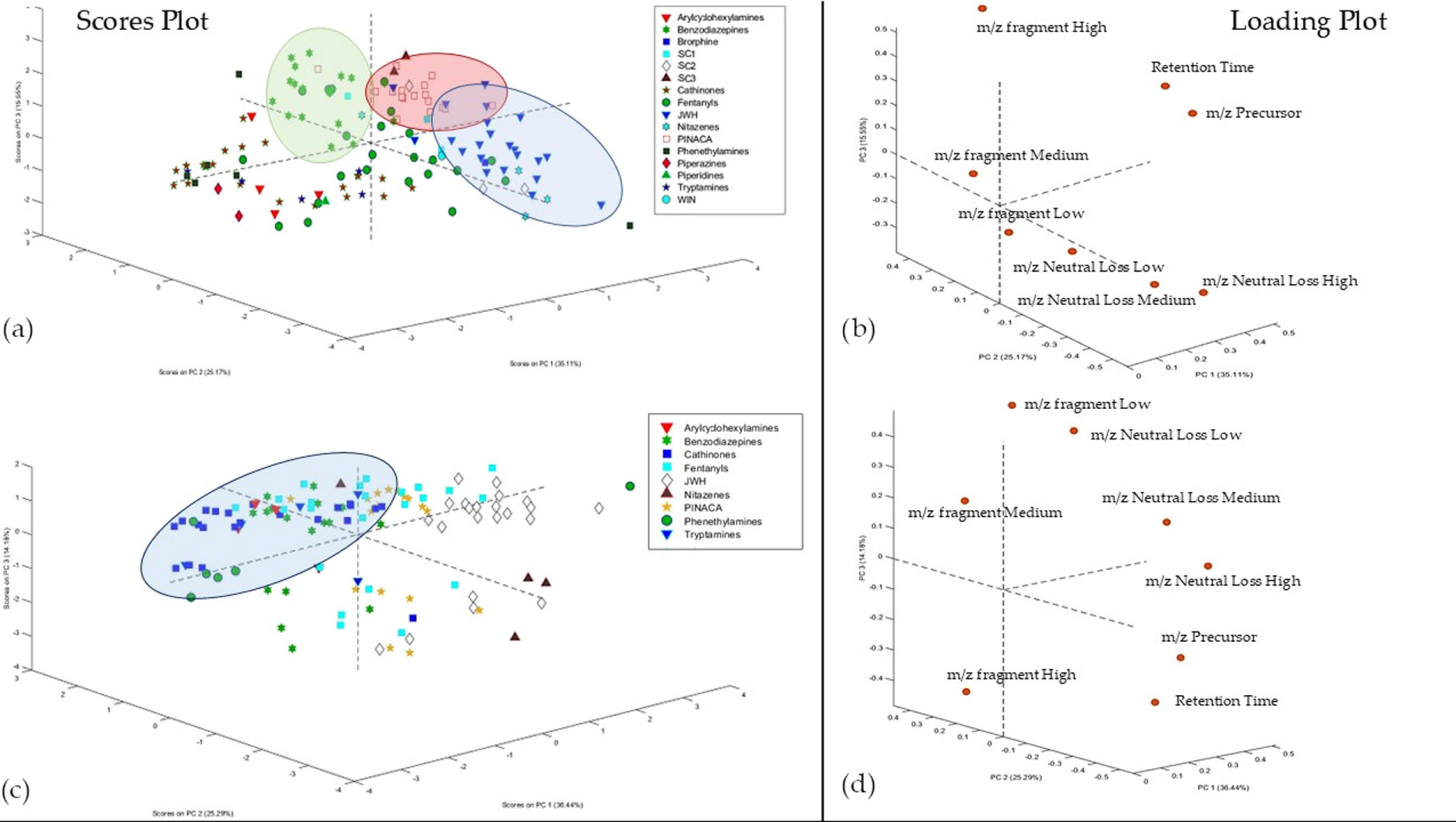
(a, b) Scores and loadings plot of the PCA calculated on the training set including all compound classes; (c, d) Scores and loadings plot of the PCA calculated on the Training set including only the nine most represented classes.

Following the graphical exploration of the data, we further subdivided the synthetic cannabinoid and synthetic opioid classes into the following respective subclasses: JWH, PINACA, WIN, SC1, SC2, and SC3 (synthetic cannabinoids), fentanyls and nitazenes (synthetic opioids). A graphical representation of the defined subclasses is shown in Figure 4, highlighting the common chemical groups.

**Figure 4:**
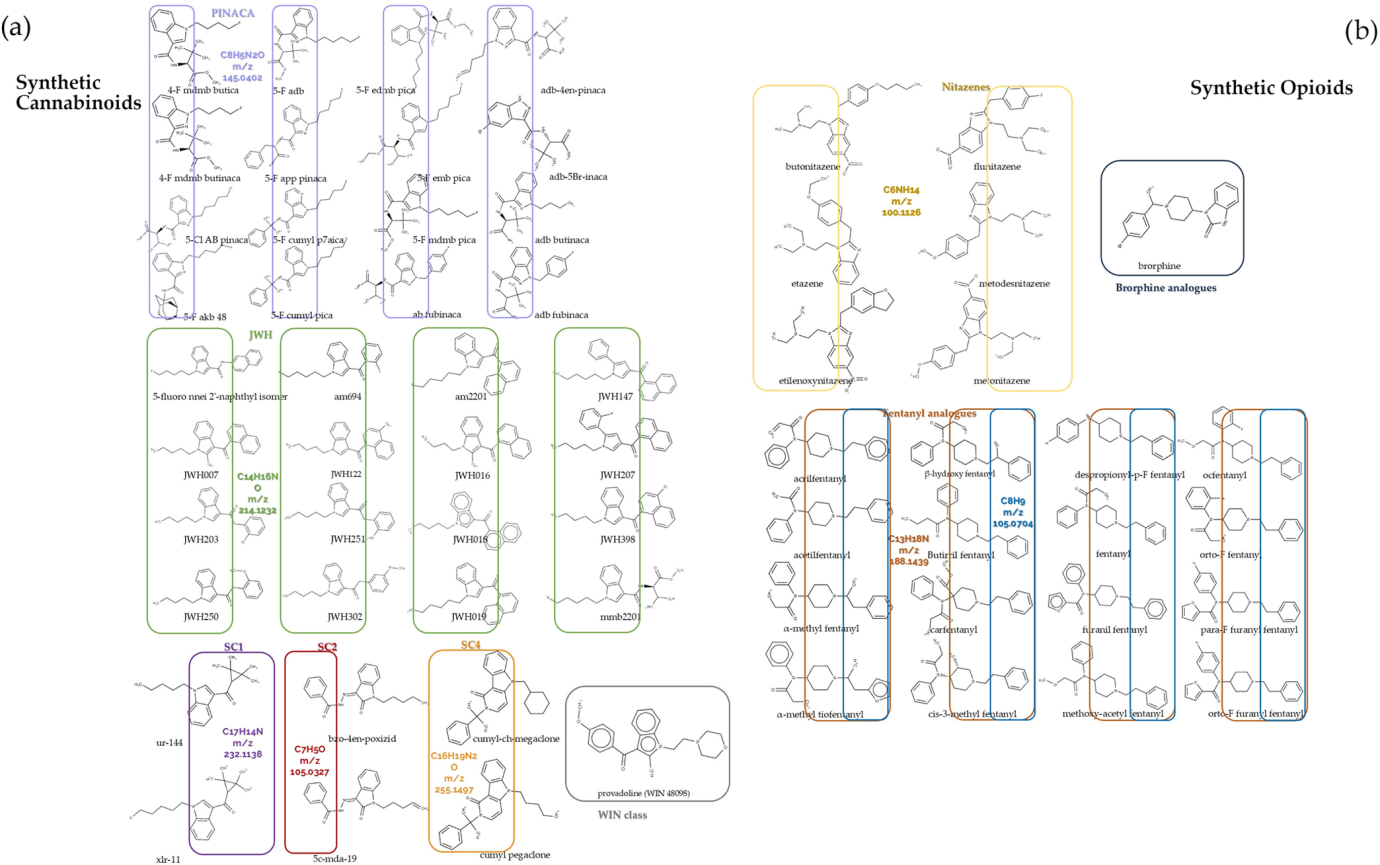
Defined subclasses within (a) synthetic cannabinoids (JWH-green, PINACA-violet, WIN-gray, SC1-purple, SC2-red, SC3-orange) and (b) synthetic opioids (brorphine analogues-blue, nitazenes-yellow, and fentanyl analogues-dark orange and blue).

With the subclassification of certain NPS, such as synthetic cannabinoids and synthetic opioids, as previously explained, it became clearer to identify which classes were more clustered together and which were less based on their distribution in the component space. The score plot clearly shows that classes such as benzodiazepines, JWH, and PINACA are more homogeneous than those like cathinones (which form two clusters, one along PC1 and the other along PC3) or fentanyls. Furthermore, the benzodiazepine class varies more along PC3, with the variables contributing positively to its differentiation from the others being the *m*/*z* of the three most intense fragments, as observed in the loading plot. The JWH class lies at negative scores along PC2, with the values of the three neutral losses contributing to its differentiation. The PINACA class, on the other hand, is mapped along positive values of both PC3 and PC1, with the variables contributing to its differentiation being the precursor *m*/*z* and retention time, which, for this class, is greater than 8 min.

A second PCA, conducted by considering only classes with at least four analytes, highlighted the cluster related to the cathinone class more distinctly (Figure 3c,d). This cluster appears more homogeneous than the first PCA and is separated from the others along Components 1 and 3. The separation along these components is driven by the *m*/*z* of the least intense fragment and its corresponding neutral loss, which helps to differentiate cathinones from other classes distinctly. In both exploratory analyses, potential outliers in the data set were identified by inspection of the values of Hotelling T-square (*T* ^2^) and Q-residuals. *T* ^2^ measures how far an observation is from the distribution of the other samples, defined as the Mahalanobis distance of the observation from the center of the scores space. Conversely, the Q-residuals capture the residual variance unexplained by the PC model. A sample is deemed not an outlier if its *T* ^2^ and Q-residual values fall below their respective critical thresholds, typically defined at a 95% confidence level. This relationship is visually represented in the influence plot.^38^

### Construction of SIMCA Classification Models

For each class, a PCA model of appropriate dimensionality is built on the training samples of the category so that classification is based on verifying whether an analyte is an outlier or not to that model. SIMCA is a supervised classification method based on creating separate models for each class of objects in the training set.^27,28^ In this work, SIMCA models were built for each class with at least four analytes; therefore, nine classes were considered. Supervised analysis was performed for the matrix acquired with a 10–30 V CE range and the one obtained within the 30–50 V range.

However, only the models derived from the first matrix are reported here, as they performed better in sensitivity and specificity. Each model was tested on the test set, as shown in the plots in Figures 5 and S1, where the projection of the samples onto the model spaces of the nine categories is displayed. The dashed curve in each graph represents the model’s acceptance threshold. The class model accepts substances falling below the threshold, while those falling above are rejected as outliers. In all cases, the classification figures of merit, particularly sensitivity and specificity, on the test set samples were generally high (Figure 6).

**Figure 5:**
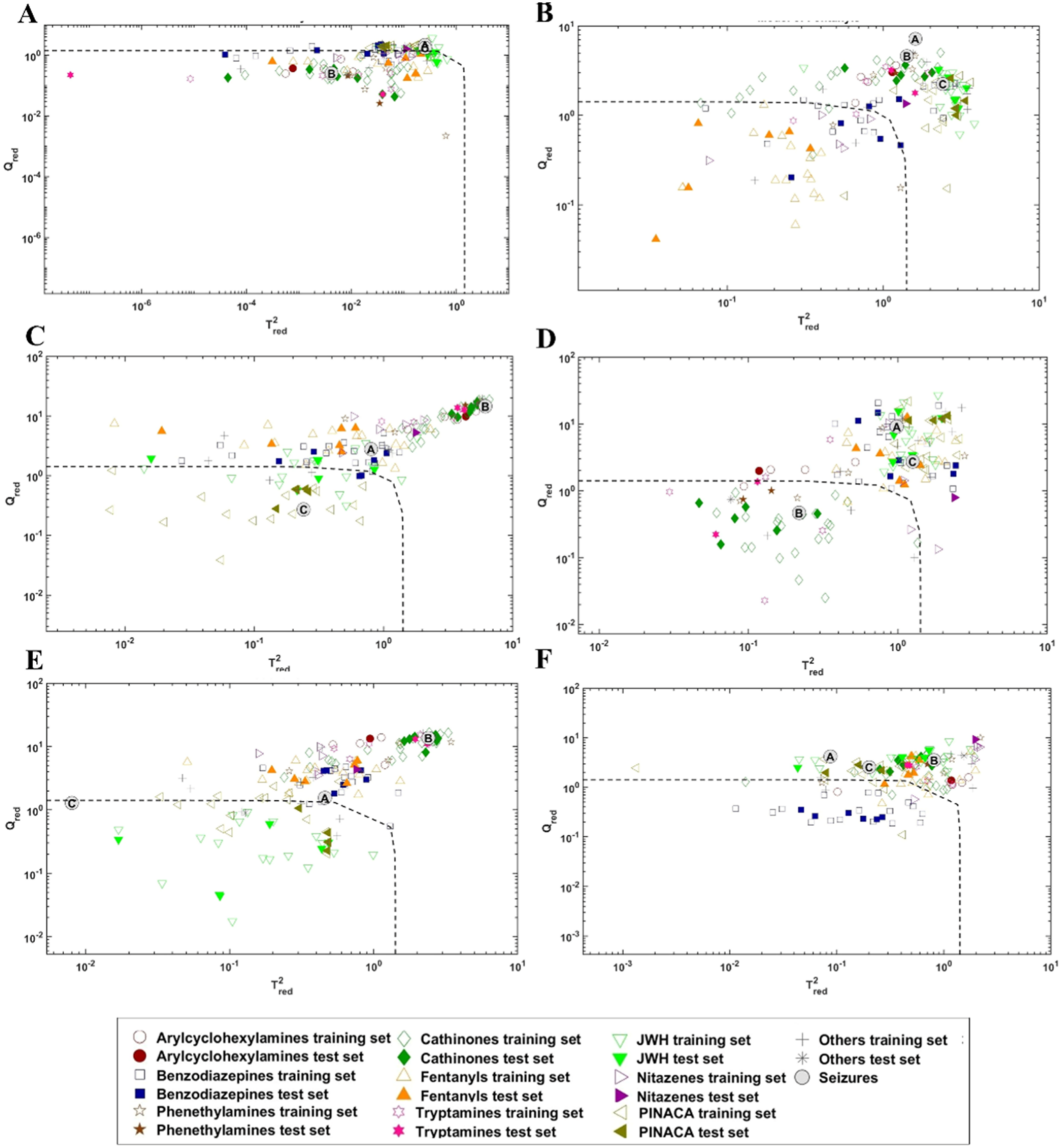
Projection of the training and test set on the SIMCA model spaces of phenethylamines (A), fentanyls (B), PINACA (C), cathinones (D), JWH (E), benzodiazepines (F) classes; model spaces of tryptamines, arylcyclohexylamines, and nitazenes are reported in Figure S1.

**Figure 6:**
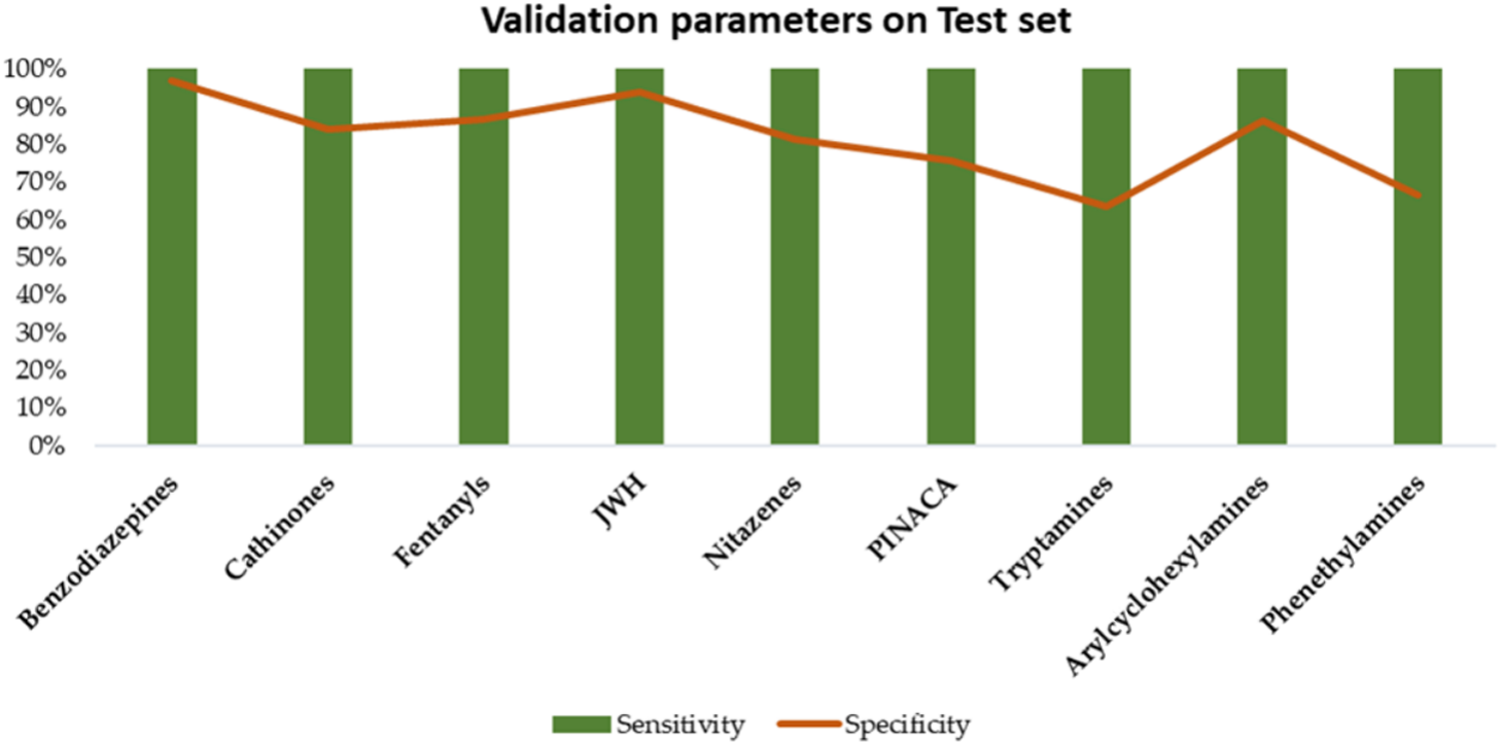
Validation parameters for each SIMCA model built for the nine classes considered (Benzodiazepines, Cathinones, Fentanyls, JWH, Nitazenes, PINACA, Tryptamines, Arylcyclohexylamines, and Phenethylamines) calculated using the training set for the CE range of 10–30 V and evaluated on the test set.

Specifically, each model achieved maximum sensitivity, correctly classifying all samples belonging to the target class. Specificity was also optimal for most classes, except for tryptamines and phenethylamines analogues, for which specificity values were 63.4% and 66.7%, respectively.

Although these values remain above 50%, the relatively lower specificity can be attributed to the limited number of analytes in their target class. The smaller sample size resulted in less robust models, making it more challenging to precisely distinguish between samples from the target and nontarget classes. This limitation is also due to reduced distances in component space, leading to greater proximity between samples from different classes and thus reducing separation effectiveness.

As previously mentioned, in the analysis conducted with a CE range of 30–50 V, the sensitivity and specificity parameters for each SIMCA model were lower, indicating reduced classification accuracy. It was observed that MS/MS spectra obtained

with CE in the 30–50 V range produced excessive fragmentation for many analytes during analysis, compromising the data quality and resulting in fewer informative MS/MS spectra. Additionally, the null data collected with the 30–50 V range represented a significant proportion of the data, making the resulting matrix less informative.

### External Validation of SIMCA Models on Real Samples

External validation was performed using three real samples. This step was essential to develop a robust and generalizable model capable of adapting to practical applications while minimizing the risk of overfitting the training set.

Each model has been validated and tested on the three aforementioned seizures, represented by the letters A, B, and C, highlighted with a circle. Based on the position of the three seizures in each graph, we can conclude that sample A was classified as not belonging to any of the target classes; this result was correct considering that this sample did not contain NPS. The model of the phenethylamine class correctly recognized sample B (identified as containing 6-APB). However, this sample was also recognized in the cathinone and tryptamine class models, suggesting a similarity between these groups. Finally, sample C matched both the JWH and PINACA classes. These results align with the chemical and structural similarities of the analyte to compounds belonging to the PINACA class, which is therefore considered its correct classification. It should be noted that the PINACA class is a subclass of synthetic cannabinoids, as are the JWH compounds.

As illustrated in Figure 4, JWH and PINACA share a common structure characterized by an indole ring linked to a long alkyl chain. This chain has the same number of methylene groups for many analytes, with a characteristic fragment corresponding to 232.1126 *m*/*z* (C_17_H_14_N), common to several analytes belonging to both classes. Consequently, misclassifications between the two subclasses may occur due to their minimal structural differences. Regarding seizure B, 6-APB was compatible with three different classes, of which only the phenethylamines classification is correct. However, it should be noted that the cathinones class shares significant structural similarities with the phenethylamines, to the extent that they are often considered as one being a subclass of the other in the literature.

The comparison between classes with structural similarities can be visualized in the Coomans’ plots in Figure 7. In a Coomans’ plot, the distances of each observation from the models of two categories are displayed and compared. The plot is divided into four distinct regions by the vertical and horizontal lines corresponding to the acceptance thresholds for the two classes.

**Figure 7:**
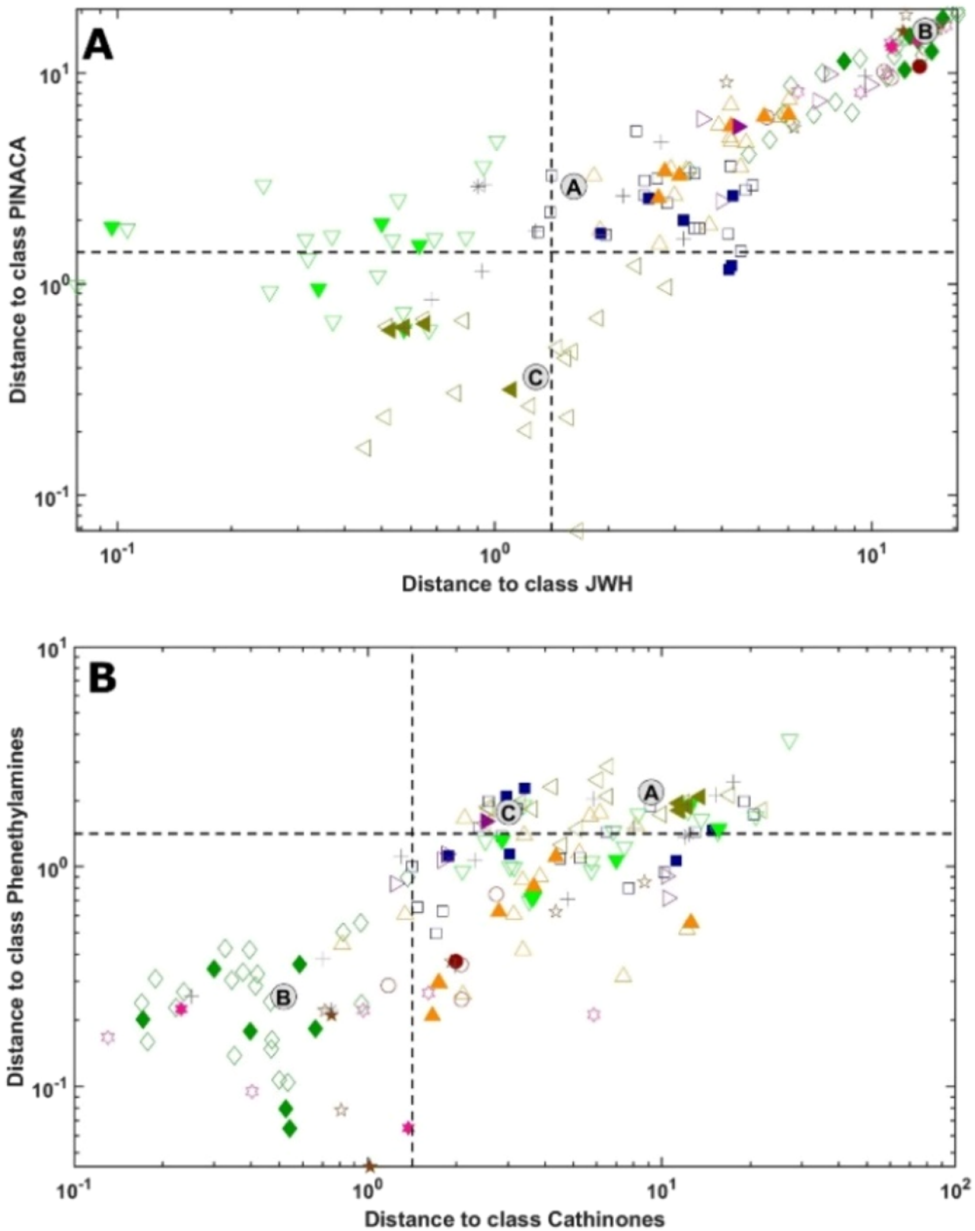
Cooman’s plots of (A) JWH and PINACA and (B) Cathinones and Phenethylamines. For the legend, refer to Figure 5.

In Figure 7a, the comparison between the JWH class and the PINACA class highlights that most of the analytes from each class are projected in the lower leftmost corner of the plot, i.e., are accepted by both class models. Sample C is also ambiguously classified; anyway, its distance to the PINACA class is lower than the distance to the JWH class, which is very close to the acceptance/rejection threshold for the category.

In Figure 7b, the comparison between the phenethylamines and cathinones classes shows a similar behavior: all the cathinones and almost all the phenethylamines are accepted by both class models, falling in the lower leftmost region of the plot. Analogously, sample B is projected onto the same area of the plot and, therefore, is accepted by both models, even if its distance to the phenethylamines class is lower.

Due to these chemical and structural similarities, misclassifications of molecules belonging to these two classes may occur. However, the important aspect of NPS monitoring is that the analyte is correctly assigned to its primary class, avoiding false negatives. Consequently, the SIMCA models for the JWH and PINACA classes, and for cathinones and phenethylamines, remain highly specific, as shown in Figure 5.

As a final point, the second reason for the incorrect classification of sample B in the tryptamine class is the low specificity of the model related to the latter, as previously highlighted (Figure 6). This is likely due to the lack of robustness of the model, which does not adequately discriminate between target and nontarget samples. This limitation is attributed to the reduced number of analytes used to build the model, resulting from the limited availability of analytical tryptamine standards.

In summary, most of the developed models were shown to be robust and may be incorporated into the HRMS data analysis workflow of any forensic laboratory dealing with unknown substances identification. Actually, LC-HRMS has been recognized as a useful tool for screening purposes in forensic toxicology, considering that the relatively long chromatographic run is compensated by its reliability and the amount of acquired information when compared to typical immunological screening tests. Data interpretation which is still a bottleneck of LC-HRMS screening may be accelerated by classification models as shown in this study.

## Conclusions

The proposed approach integrates advanced analytical strategies based on UPLC-HRMS technologies with multivariate analysis to monitor the rapid emergence of NPS. The supervised SIMCA approach proved effective in correctly identifying the class of various compounds, even in real contexts, as highlighted by the analysis of real samples. External validation conducted on three real samples confirmed the models’ ability to generalize and adapt to data not included in the training set, reducing the risk of overfitting and demonstrating strong predictive performance, as evidenced by the positive validation parameters obtained for each model.

Despite the overall positive results, some cases of misclassification emerged. An example concerns the tryptamine model, which showed relatively low specificity, leading to false positives attributable to the scarcity of available standards in this class, thereby limiting the model’s robustness.

To further improve performance and minimize classification errors, continuous model updates are essential, including a greater number of analytes for each class. In parallel, increasing the number of descriptive variables in the data set could enhance the model’s ability to more precisely differentiate analytes based on their physicochemical similarities.

A key advantage of the SIMCA approach lies in its flexibility. Unlike other techniques, such as PLS-DA, which attempt to model all classes within a single model and require known samples, SIMCA constructs separate models for each class. This feature eliminates the need for samples from nontarget classes, allowing the possibility of not classifying samples into any of the modeled categories. This multivariate approach proves particularly useful for classifying unknown NPS structurally related to those already known identifying potential new classes and recognizing metabolites, paving the way for future applications in analyzing biological matrices. This capability represents an added value in the context of NPS monitoring, increasing the knowledge of this constantly evolving phenomenon and providing strong support for forensic investigations. In addition, the approach described can be valuable in other fields, such as doping analysis, metabolomics, and environmental analysis.

## Supplementary Material



## Notes

NPS data set; the first part is the training set (T), and the second is the validation set (V) (Table S1); projection of the training and test set on the SIMCA model spaces of tryptamines (G), arylcyclohexylamines (H), and nitazenes (I) (Figure S1) (PDF)
