# Supplementary material for: Soft Independent Modeling of Class Analogies for the Screening of New Psychoactive Substances through UPLC-HRMS/MS
Source: Anal Chem. 2025 Jul 11;97(28):15420–9. doi: 10.1021/acs.analchem.5c02450 (PMC12291040; doi:10.1021/acs.analchem.5c02450)
Supplement: Supplementary file 1 [file ac5c02450_si_001.pdf]

Supporting information

Soft Independent Modeling of Class Analogies for the screening of New Psychoactive Substances through UPLC-HRMS/MS

Ilenia Bracaglia[1,2], Sara Gamberoni[1], Camilla Montesano[1]*, Francesco Bartolini[1], Sabino Napoletano[1,3], Claudio D'Alfonso[3], Chiara Nieri[3], Federico Marini[1], Manuel Sergi[1]

[1]Department of Chemistry, Sapienza University of Rome, 00185 Rome, Italy

[2]Department of Public Health and Infectious Diseases, Sapienza University of Rome, 00185 Rome, Italy

[3]Department of Public Security, Directorate for the Forensic Science Police and the Cyber Security, Forensic Science Police Service, 00173 Rome, Italy

*Correspondence to: camilla.montesano@uniroma1.it

Table of contents

Tab S1………………………………………………………………………………………………….S3

Fig S1…………………………………………………………………………………………………..S7

Table S1: NPS dataset; the first part is the training set (T), and the second is the validation set (V). The class group is assigned based on the classification found in the HighResNPS database[11].

Drug name	Drug Class	Set	Precursor Ion (m/z)	Rt (min)	m/z 1	m/z 2	m/z 3	m/z Neutral Loss 1	m/z Neutral Loss 2	m/z Neutral Loss 3
3-OH-PCP	Arylciclohexylamine	T	260.201	4.03	107.049	175.112	0	153.152	85.089	0
Pcp	Arylciclohexylamine	T	244.206	7.96	219.117	204.094	232.113	25.089	40.113	12.093
Ketamine	Arylciclohexylamine	T	238.099	3.14	125.016	220.089	179.063	113.084	18.011	59.037
Deschloro-N-Ethyl-Ketamine	Arylciclohexylamine	T	218.154	3.12	91.055	173.097	145.102	127.099	45.058	73.053
Nimetazepam	Benzodiazepine	T	296.103	8.92	250.098	221.108	0	46.005	74.996	0
Oxazepam	Benzodiazepine	T	287.059	7.85	241.053	269.048	243.045	46.005	18.011	44.014
Lorazepam	Benzodiazepine	T	321.019	8.27	275.014	277.006	303.009	46.005	45.986	18.011
Bromazepam	Benzodiazepine	T	316.007	6.59	209.071	182.084	289.923	106.937	133.924	26.086
Prazepam	Benzodiazepine	T	325.111	11.6	271.064	140.095	0	54.047	185.016	0
Diclazepam	Benzodiazepine	T	319.067	11.2	291.048	218.073	154.033	28.019	100.994	165.034
Clonazolam	Benzodiazepine	T	354.076	7.52	308.070	326.057	310.062	46.005	28.019	44.014
Diclazepam	Benzodiazepine	T	319.040	10.8	227.050	154.042	291.022	91.990	164.998	28.019
Diazepam	Benzodiazepine	T	285.079	10.5	193.089	222.092	257.061	91.990	62.987	28.019
Alprazolam	Benzodiazepine	T	309.091	8.54	281.072	274.029	205.066	28.018	35.061	104.025
Bentazepam	Benzodiazepine	T	297.106	7.65	166.069	269.087	241.077	131.037	28.019	56.029
Nitrazepam	Benzodiazepine	T	282.088	7.77	236.082	207.092	180.081	46.005	74.996	102.007
Flualprazolam	Benzodiazepine	T	327.081	10.5	299.063	0	0	28.019	0	0
Medazepam	Benzodiazepine	T	271.100	8.11	207.105	0	0	63.995	0	0
Flurazepam	Benzodiazepine	T	388.159	6.68	315.070	317.073	288.059	73.089	71.086	100.100
Fluetizolam	Benzodiazepine	T	327.108	8.62	298.069	273.086	0	29.039	54.022	0
Tetrazepam	Benzodiazepine	T	289.111	11.3	253.134	197.120	225.103	35.977	91.990	64.008
Brorphine	Brorphine	T	400.102	6.37	218.129	182.981	184.971	181.973	217.122	215.131
1-Naphyrone	Cathinone	T	282.186	6.89	141.070	211.112	127.055	141.115	71.074	156.057
Pentylone	Cathinone	T	236.129	3.72	188.107	218.118	175.063	48.021	18.011	61.065
3-Chloromethcathinone	Cathinone	T	198.069	3.38	145.089	180.059	0	52.979	18.011	0
2-Methyl-α-Pyrrolidinopropiophenone	Cathinone	T	218.155	3.61	119.086	147.081	98.097	99.068	71.073	120.057
α-Pvp	Cathinone	T	232.170	4.19	126.128	91.055	161.097	106.042	141.115	71.073
α-Pyrrolidinohexanophenone	Cathinone	T	246.186	5.55	140.145	91.055	105.034	106.042	155.131	141.152
N-Cyclohexyl Butylone	Cathinone	T	290.176	5.83	160.076	190.087	132.079	130.099	100.089	158.097
Methcathinone	Cathinone	T	164.108	1.83	146.097	131.073	105.070	18.011	33.034	59.037
Butylone	Cathinone	T	222.113	2.55	174.092	204.102	146.094	48.021	18.011	76.019
3.4-Dimethylmethcathinone	Cathinone	T	192.139	4.11	159.105	174.128	144.081	33.034	18.011	48.057
4-Chloro-α-Pvp	Cathinone	T	266.131	6.18	125.016	195.058	138.995	141.115	71.073	127.136
Mdpv	Cathinone	T	276.160	4.45	201.043	234.126	249.149	75.117	42.034	27.011
3,4-MD-α-Php	Cathinone	T	290.176	5.68	135.045	140.144	189.092	155.129	150.029	101.084
3-Methylmethcathinone	Cathinone	T	178.123	1.86	160.113	132.081	0	18.011	46.042	0
3,4-Trimethylene-α-Piperidinovalerophenone	Cathinone	T	286.217	8.09	131.086	201.128	140.144	155.131	85.089	146.073
Dimethylcathinone	Cathinone	T	178.123	1.93	133.065	144.081	0	45.058	34.042	0
N-Butyl Butylone	Cathinone	T	264.160	5.02	246.149	216.139	174.092	18.011	48.021	90.068
Methedrone	Cathinone	T	194.118	2.37	176.108	161.084	146.061	18.011	33.034	48.058
B-Pentedrone	Cathinone	T	192.139	3.48	174.128	132.081	144.081	18.011	60.058	48.058
N-Ethyl Hexedrone	Cathinone	T	220.170	5.26	202.159	146.097	130.066	18.011	74.073	90.104

Diethylcathinone	Cathinone	T	206.154	2.75	163.055	105.070	133.065	43.097	101.084	73.089
Etazene	Cathinone	T	352.239	5.48	100.113	0	0	252.129	0	0
Xlr-11	CS1	T	330.223	12.07	232.114	125.097	312.213	98.109	205.127	18.011
Ur-144	CS1	T	312.233	12.3	214.123	294.222	279.199	98.109	18.011	33.034
Ab005	CS2	T	353.259	9.39	112.113	256.170	125.097	241.147	97.089	228.163
Bzo-4En-Poxizid	CS2	T	334.156	11.7	105.033	77.039	0	229.123	257.116	0
5-Fluoro Pb-22	CS2	T	377.166	11.6	232.113	0	0	145.054	0	0
Cumyl Pegaclone	CS3	T	373.228	11.9	255.149	0	0	118.078	0	0
Cumyl-Ch-Megaclone	CS3	T	399.244	12.1	281.165	0	0	118.078	0	0
Para-Fluoro Furanyl Fentanyl	Fentanyl analogue	T	393.198	6.59	188.144	0	0	205.054	0	0
Cis 3-Methyl Thiofentanyl	Fentanyl analogue	T	357.200	6.65	208.116	160.113	230.154	149.084	197.087	127.046
Remifentanil	Fentanyl analogue	T	377.208	4.69	317.186	285.160	228.124	600.212	92.047	149.084
Carfentanil	Fentanyl analogue	T	395.234	7.13	335.212	246.149	279.186	60.021	171.112	138.075
2-Methyl AP-237	Fentanyl analogue	T	287.212	5.98	117.070	0	0	170.142	0	0
Ocfentanil	Fentanyl analogue	T	371.213	5.18	188.144	105.070	0	183.069	266.143	0
Despropionyl Para-Fluorofentanyl	Fentanyl analogue	T	299.192	6.57	188.144	105.070	0	111.049	194.122	0
Acryl Fentanyl	Fentanyl analogue	T	335.212	6.02	188.144	0	0	147.068	0	0
Orthofluoro Fentanyl	Fentanyl analogue	T	355.219	6.17	259.181	208.114	188.144	96.037	147.105	167.075
Acetyl Fentanyl	Fentanyl analogue	T	323.212	5.13	188.144	105.070	202.123	135.068	218.142	121.089
Butyryl Fentanyl	Fentanyl analogue	T	351.244	6.99	188.144	230.154	202.159	163.099	121.089	149.084
2'-Fluoro-Ortho-Fluoro-Cis-3-Methyl Fentanyl	Fentanyl analogue	T	387.225	7.69	220.151	123.062	248.144	167.075	264.163	139.081
Sufentanil	Fentanyl analogue	T	387.211	7.74	238.127	355.184	206.100	149.084	32.026	181.110
Ah7921	Fentanyl analogue	T	329.119	6.75	284.061	286.064	172.956	45.058	43.055	156.163
Beta-Hydroxy Fentanyl	Fentanyl analogue	T	353.223	5.41	335.212	188.144	204.139	18.011	165.079	149.084
α-Methyl Fentanyl	Fentanyl analogue	T	351.244	5.41	188.144	202.159	91.055	163.099	149.084	260.189
Alfentanil	Fentanyl analogue	T	417.261	6.38	165.103	268.177	197.129	252.159	149.084	220.132
Fentanyl	Fentanyl analogue	T	337.228	6.21	105.070	188.144	216.139	232.157	149.084	121.089
Rcs-4	JWH	T	322.181	11.8	135.045	0	0	187.136	0	0
Jwh-307	JWH	T	386.192	12.1	155.049	127.055	0	231.142	259.137	0
Jwh-016	JWH	T	342.186	12.0	155.049	127.055	214.123	187.136	215.131	128.063
Rcs-8	JWH	T	376.228	12.2	121.065	91.055	144.081	255.162	285.173	232.146
Jwh-251	JWH	T	320.201	11.6	214.123	105.070	144.045	106.078	215.131	176.156
Cb-13	JWH	T	369.185	12.8	171.045	155.049	299.107	198.141	214.136	70.078
Jwh-018	JWH	T	342.186	12.1	155.049	214.123	127.055	187.136	128.063	215.131
Jwh-098	JWH	T	386.212	12.2	185.039	228.139	0	201.173	158.073	0
Mmb2201	JWH	T	363.209	11.4	232.113	0	0	131.095	0	0
Jwh-203	JWH	T	340.147	11.9	125.016	214.123	188.144	215.131	126.024	152.003
Mam2201	JWH	T	374.192	11.9	169.065	232.113	355.174	206.167	142.079	19.018
Am-694	JWH	T	436.057	11.7	230.931	309.052	202.936	205.127	127.005	233.122
Bb-22	JWH	T	385.192	12.1	240.139	144.045	0	145.053	241.147	0
Am-2201	JWH	T	360.176	11.8	232.113	127.055	155.049	128.063	233.122	205.127
Am-2233	JWH	T	459.093	7.46	362.004	230.931	112.113	97.089	228.163	346.981
Jwh-398	JWH	T	376.147	12.3	189.011	191.014	214.123	187.136	185.133	162.024
Jwh-250	JWH	T	336.196	11.9	121.065	91.055	200.144	215.131	245.142	136.053
Metonitazene	Nitazene	T	383.208	5.81	100.113	121.065	0	283.096	262.143	0
Butonitazene	Nitazene	T	425.255	9.41	100.113	0	0	325.143	0	0
Etonitazepyne	Nitazene	T	395.208	8.52	98.097	165.070	0	297.111	230.138	0
Flunitazene	Nitazene	T	371.188	6.08	252.106	298.099	253.109	119.082	73.089	118.079
2-CI	Phenethylamine	T	308.015	5.09	290.988	275.965	0	17.026	32.050	0

5-Eapb	Phenethylamine	T	204.139	4.13	131.049	159.081	0	73.089	45.058	0
5-Mmpa	Phenethylamine	T	170.100	3.26	139.056	0	0	310.445	0	0
5-Mapb	Phenethylamine	T	190.123	3.54	159.081	131.049	0	31.042	59.073	0
25I Nbome	Phenethylamine	T	428.072	8.66	121.065	91.055	272.141	307.007	337.017	155.931
2-CH	Phenethylamine	T	182.118	2.73	138.068	165.092	135.052	45.058	17.026	47.066
5-Fluoro Edmb-Pica	PINACA	T	391.239	11.7	232.113	0	0	1.591.271	0	0
5-Fluoro App Pinaca	PINACA	T	397.204	10.9	233.116	352.189	368.190	164.088	45.014	29.013
4-Fluoro Mdmb-Butinaca	PINACA	T	364.204	11.6	219.093	304.184	145.041	145.110	60.019	219.162
5-Fluoro Mdmb-7-Paica	PINACA	T	378.219	11.3	318.199	233.109	298.192	60.019	145.110	80.027
Adb-4En-Pinaca	PINACA	T	343.213	11.2	213.103	298.192	0	130.111	45.021	0
Mdmb-4En-Pica	PINACA	T	357.218	11.7	212.107	0	0	145.110	0	0
5-Fluoro Akb-48	PINACA	T	384.245	12.4	240.139	135.117	0	144.106	249.128	0
Cumyl-Nbminaca	PINACA	T	388.239	12.2	253.134	270.161	0	135	118.078	0
5-Fluoro Cumyl-P7AICA	PINACA	T	368.214	11.3	250.136	230.129	174.067	118.078	138.084	194.147
Mdmb-4En-Pinaca	PINACA	T	358.213	11.9	213.103	298.192	0	145.110	60.021	0
5-Cloro Ab-Pinaca	PINACA	T	365.174	10.9	249.079	320.153	213.103	116.095	45.021	152.072
Ab-Fubinaca	PINACA	T	369.173	10.2	253.078	324.159	109.040	116.095	45.014	260.127
5-Fluoro Cumyl-Pica	PINACA	T	367.219	11.6	249.140	206.134	232.113	118.078	161.084	135.106
Adb-5Br Inaca	PINACA	T	353.061	8.21	310.036	308.041	222.951	43.026	45.020	130.111
Adb-Fubinaca	PINACA	T	383.189	11.1	253.078	338.167	109.041	130.111	45.021	274.147
5-Fluoro Adb	PINACA	T	378.219	11.7	233.109	318.199	213.103	145.110	60.019	165.116
Adb-Butinaca	PINACA	T	331.213	11.2	201.103	286.192	145.040	130.111	45.022	186.173
1,4 Methoxy Phenyl Piperazine	Piperazine	T	193.134	2.17	150.092	119.073	133.053	43.042	74.041	600.813
Benzyl Piperazine	Piperazine	T	177.139	2.22	91.055	85.077	0	86.084	92.063	0
Ethylphenidate	Piperidine	T	248.165	5.03	84.081	174.128	0	164.084	74.037	0
4-Hydroxy Dmt	Tryptamine	T	205.134	3.38	160.076	115.055	132.081	45.058	90.079	73.053
4-Acetoxy Dipt	Tryptamine	T	303.207	3.68	146.097	182.118	189.139	157.110	121.089	114.068
N.N - Dmt	Tryptamine	T	189.139	2.36	144.081	174.128	0	45.058	15.011	0
5-Meo Mipt	Tryptamine	T	247.181	3.70	121.065	175.112	159.081	126.116	72.069	88.100
5-Methoxy Dpt	Tryptamine	T	275.212	5.38	174.092	114.128	0	129.115	189.079	0
Provadoline (Win 48,098)	WIN	T	379.202	8.29	135.045	0	0	244.158	0	0
Methoxetamine	Arylciclohexylamine	V	248.165	3.79	121.065	203.107	159.079	127.099	45.058	89.084
Triazolam	Benzodiazepine	V	343.052	8.67	308.039	315.020	310.006	35.061	28.031	33.045
Etizolam	Benzodiazepine	V	343.078	9.25	314.039	316.039	307.101	29.039	27.024	35.977
Midazolam	Benzodiazepine	V	326.086	7.11	291.073	244.044	209.066	35.013	82.042	117.020
Lormetazepam	Benzodiazepine	V	335.035	9.57	289.029	243.069	0	46.006	91.967	0
Flunitrazepam	Benzodiazepine	V	314.094	8.67	268.089	239.099	0	46.005	74.995	0
Estazolam	Benzodiazepine	V	295.075	7.78	267.056	269.059	205.077	28.019	26.016	89.998
N-ethyl pentedrone	Cathinone	V	206.154	5.03	188.144	146.097	130.066	18.011	60.058	76.089
3-methilmethcathinone	Cathinone	V	178.123	3.05	160.113	147.081	144.081	18.011	31.042	34.042
4-fluoromethcathinone	Cathinone	V	182.098	2.23	164.039	149.061	0	18.059	33.037	0
4-methylethcathinone	Cathinone	V	192.139	3.05	174.128	145.089	119.086	18.011	47.049	73.053

Eutylone	Cathinone	V	236.129	3.06	188.108	218.118	174.055	48.021	18.011	62.073
Ethylone	Cathinone	V	222.113	2.19	174.092	204.103	146.094	48.021	18.011	76.019
4-chloromethcathinone	Cathinone	V	198.069	3.38	145.089	180.057	0	52.979	18.011	0
5c-mda-19	CS2	V	336.171	11.9	105.033	770.391	0	2.311.385	259.132	0
α-methyl thiofentanyl	Fentanyl	V	357.200	6.56	208.116	160.113	230.155	149.084	197.088	127.046
Furanil fentanyl	Fentanyl	V	375.207	6.47	188.144	105.070	0	187.063	270.137	0
Cis-3-methyl fentanyl	Fentanyl	V	351.244	6.64	188.144	202.159	91.055	163.099	149.084	260.189
Methoxyacetyl fentanyl	Fentanyl	V	353.223	4.94	188.144	0	0	165.079	0	0
Parafluoro fentanyl	Fentanyl	V	355.219	6.17	259.181	208.114	188.144	96.038	147.105	167.075
Jwh-302	JWH	V	336.196	11.8	214.123	121.065	188.144	122.073	215.131	148.053
Jwh-147	JWH	V	382.217	12.3	155.049	127.055	0	227.167	255.162	0
Jwh-007	JWH	V	356.201	12.1	155.049	127.055	228.139	201.152	229.147	128.063
Jwh-122	JWH	V	356.201	12.3	169.065	214.123	141.070	187.136	142.078	215.131
Jwh-019	JWH	V	356.201	12.2	155.049	127.055	228.139	201.152	229.147	128.063
5-fluoro nnei 2'-naphthyl isomer	JWH	V	375.187	11.7	232.113	0	0	143.075	0	0
Metodesnitazene	Nitazene	V	338.223	4.42	100.113	0	0	238.111	0	0
Ethyleneoxynitazene	Nitazene	V	395.208	6.55	100.113	0	0	295.096	0	0
6-mapb	Phenethylamine	V	190.123	3.54	159.081	131.049	0	31.042	59.073	0
Mda	Phenethylamine	V	180.102	2.93	135.045	163.076	133.065	45.058	17.027	47.037
5-fluoro mdmb-pica	PINACA	V	377.224	11.5	232.113	0	0	145.110	0	0
5-fluoro emb-pica	PINACA	V	377.224	11.6	232.113	0	0	145.110	0	0
Mdmb chmica	PINACA	V	385.249	12.0	240.139	0	0	145.110	0	0
4-fluoro mdmb-butica	PINACA	V	363.208	11.6	218.097	0	0	145.110	0	0
1,4 fluoro phenyl piperazina	Piperazine	V	181.114	2.62	138.072	136.056	150.072	43.042	45.058	31.042
5-methoxy amt	Tryptamine	V	205.134	3.38	163.087	135.056	105.070	42.047	70.078	100.064
5-methoxy dmt	Tryptamine	V	219.149	2.54	174.092	159.068	0	45.058	60.081	0

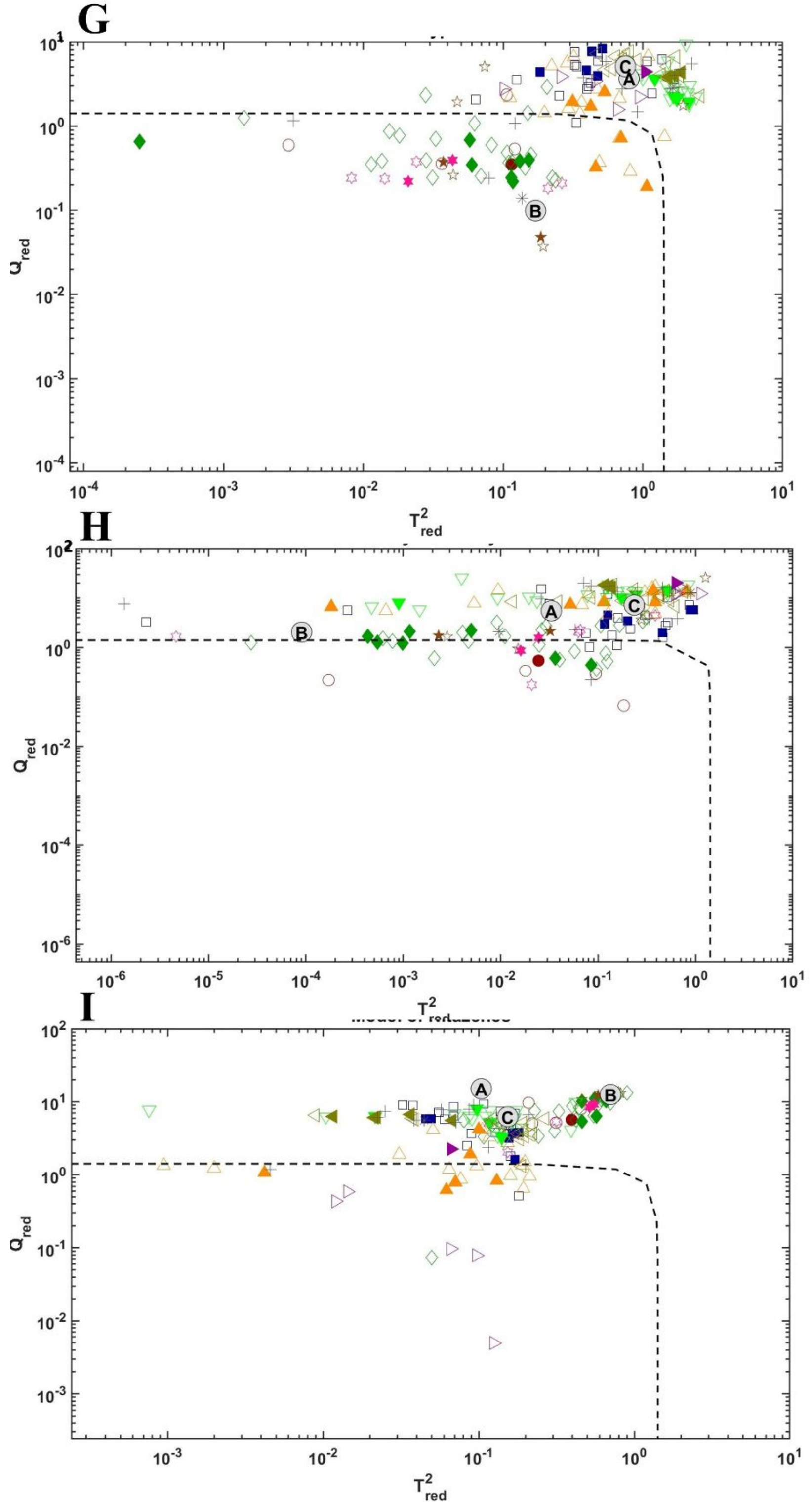

Fig S1. Projection of the training and test set on the SIMCA model spaces of tryptamines (G), arylcyclohexylamines (H), and nitazenes (I)
